# Modeling Insertional Mutagenesis Using Gene Length and Expression in Murine Embryonic Stem Cells

**DOI:** 10.1371/journal.pone.0000617

**Published:** 2007-07-18

**Authors:** Alex S. Nord, Karen Vranizan, Whittemore Tingley, Alexander C. Zambon, Kristina Hanspers, Loren G. Fong, Yan Hu, Peter Bacchetti, Thomas E. Ferrin, Patricia C. Babbitt, Scott W. Doniger, William C. Skarnes, Stephen G. Young, Bruce R. Conklin

**Affiliations:** 1 Department of Medicine, MacDonald Medical Research Laboratories, University of California at Los Angeles, California, United States of America; 2 Gladstone Institute of Cardiovascular Disease, San Francisco, California, United States of America; 3 Departments of Biopharmaceutical Sciences and Pharmaceutical Chemistry, University of California at San Francisco, California, United States of America; 4 Department of Medicine, University of California at San Francisco, California, United States of America; 5 Functional Genomics Laboratory, University of California at Berkeley, California, United States of America; 6 Department of Epidemiology and Biostatistics, University of California at San Francisco, California, United States of America; 7 Washington University School of Medicine, St. Louis, Missouri, United States of America; 8 Wellcome Trust Sanger Institute Hinxton, Cambridge, United Kingdom; 9 Department of Molecular and Cellular Pharmacology, University of California at San Francisco, California, United States of America; Pasteur Institute, France

## Abstract

**Background:**

High-throughput mutagenesis of the mammalian genome is a powerful means to facilitate analysis of gene function. Gene trapping in embryonic stem cells (ESCs) is the most widely used form of insertional mutagenesis in mammals. However, the rules governing its efficiency are not fully understood, and the effects of vector design on the likelihood of gene-trapping events have not been tested on a genome-wide scale.

**Methodology/Principal Findings:**

In this study, we used public gene-trap data to model gene-trap likelihood. Using the association of gene length and gene expression with gene-trap likelihood, we constructed spline-based regression models that characterize which genes are susceptible and which genes are resistant to gene-trapping techniques. We report results for three classes of gene-trap vectors, showing that both length and expression are significant determinants of trap likelihood for all vectors. Using our models, we also quantitatively identified hotspots of gene-trap activity, which represent loci where the high likelihood of vector insertion is controlled by factors other than length and expression. These formalized statistical models describe a high proportion of the variance in the likelihood of a gene being trapped by expression-dependent vectors and a lower, but still significant, proportion of the variance for vectors that are predicted to be independent of endogenous gene expression.

**Conclusions/Significance:**

The findings of significant expression and length effects reported here further the understanding of the determinants of vector insertion. Results from this analysis can be applied to help identify other important determinants of this important biological phenomenon and could assist planning of large-scale mutagenesis efforts.

## Introduction

Complete collections of well-defined mutants have helped shed light on the biology of model organisms, such as flies [1]–[3] and bacteria [4], [5]. Likewise, the development of a complete collection of mouse mutants would enhance our ability to understand mammalian biology [6]. Libraries of mutant mouse embryonic stem cells (ESCs) are particularly valuable because they can be readily cryopreserved and used to generate mutant mice. Gene trapping in ESCs is an effective, high-throughput technique for generating insertional mutations in the mouse genome [7]. Ultimately, however, non-targeted trapping becomes inefficient; some genes are repeatedly trapped, and others are trapped rarely, if at all [8], [9]. A better understanding of the characteristics that determine susceptibility (or resistance) to trapping would be useful, as it would further understanding of vector insertion into the genome and could help guide large-scale mouse mutagenesis efforts.

The factors that determine the “trappability” of individual genes (*i.e.*, their likelihood of being inactivated by gene trapping) are unclear. The integration of gene-trapping vectors into chromosomal DNA is potentially influenced by a number of factors, including the intrinsic properties of the vector, the expression level of the gene in mouse ESCs, chromatin structure, DNA substrate recognition, and gene size. In addition the existence of highly favored integration sites (hotspots) complicates efforts to understand the factors that control trappability. [10]

Gene expression levels in ESCs are believed to positively correlate with trapping efficiency with expression-dependent vectors, but the extent of the expression effect in different gene-trap vectors has not been systematically quantified or compared. Splice-acceptor (SA) gene-trap vectors depend on the integration of a new SA sequence to interrupt the trapped gene [11], [12]. When successful, SA-trap vectors inactivate the trapped gene and result in an antibiotic-resistance gene product that allows for selection of the mutant cell lines. These vectors lack a promoter, so endogenous gene expression is required to drive transcription of the vector product. However, gene expression has not been tested on a large scale while controlling for gene length, which is also thought to affect trappability.

In polyadenylation (poly-A) gene-trap vectors, by contrast, the antibiotic-resistance gene is driven by a strong promoter within the vector. The stability of the transcript for the antibiotic-resistance gene depends on the poly-A signal from the trapped gene [13]. Because the transcription of the antibiotic-resistance gene product does not depend on the endogenous expression of the trapped gene, poly-A trap vectors are predicted to trap genes regardless of whether the gene is expressed in ESCs.

The method of vector delivery to cells (retroviral vector versus plasmid DNA) may also influence which genes are inactivated by gene trapping. Retroviruses are predicted to insert at the 5′ end of transcriptionally active genes and may recognize specific substrates in genomic DNA. Little is known about the insertion of plasmid vectors. Both plasmid and retroviral methods have been used in SA gene trapping, while poly-A approaches have exclusively used retroviral delivery methods.

The recent release of a near-complete mouse genome, advances in techniques for estimating the levels of gene expression in a cell, and the availability of a public gene-trapping database (www.genetrap.org) make it possible to globally assess the likelihood that a gene will be inactivated by gene trapping. In this study, we used regression techniques to model the effects of gene length and gene-expression levels on gene trapping in different gene-trap vectors. We also sought to define hotspots for gene-trapping events by using the regression models to identify genes trapped more frequently than predicted by the models. Our findings provide an improved understanding of the factors that control vector insertion in the genome.

## Results and Discussion

### Association of gene expression and length with gene-trap likelihood

We sought to formally test the hypothesis that length and/or expression influence the probability that a gene will be trapped. We applied stringent criteria to the genes included in this analysis, limiting the dataset to genes for which accurate genomic mapping and curated annotation were available. Because absolute gene expression estimates, as opposed to fold changes, were necessary for this analysis, we employed Affymetrix Gene Chips and the GCRMA (GeneChip Robust Multi-array Analysis) gene expression estimation method (http://www.bioconductor.org) on a representative sample of E14 mouse embryonic stem cells. GCRMA expression estimates were validated by comparisons to RT-PCR data in the same E14 mouse ESC line [10] (Table S1). The correlation between GCRMA and the RT-PCR-derived expression was high (Spearman's *r* = 0.67, P-value<.0001), and the relationship between expression and gene-trap likelihood in endogenous expression-dependent vectors is consistent with previous analysis of trap likelihood with SA-plasmid vectors [10]. This level of quality control and validation gave us confidence that we accurately assessed relative gene expression throughout the full range of transcriptional activity.

**Figure 1:**
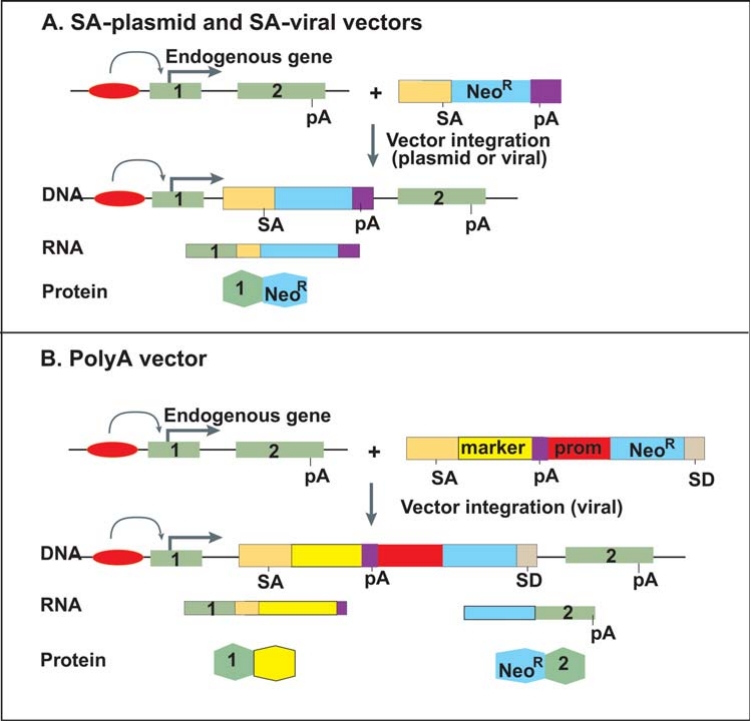
Diagram of major mechanisms of gene trapping of an endogenous gene with two exons. (A) In the SA-trap, the SA site allows trapping when inserted into any part of the gene *via* plasmid or viral integration. (B) The poly-A trap relies on the poly-A (pA) of the endogenous gene because the neomycin-resistance gene does not have a poly-A tail. Note that the poly-A trap has its own constitutive promoter (prom). Also indicated are the splice donor (SD), splice acceptor (SA), and neomycin resistance (NeoR). The major components of each trap were excluded from this diagram to emphasize on the essential elements needed to understand the trapping models. Detailed maps of each major vector type are referenced in the Methods section.

**Figure 2:**
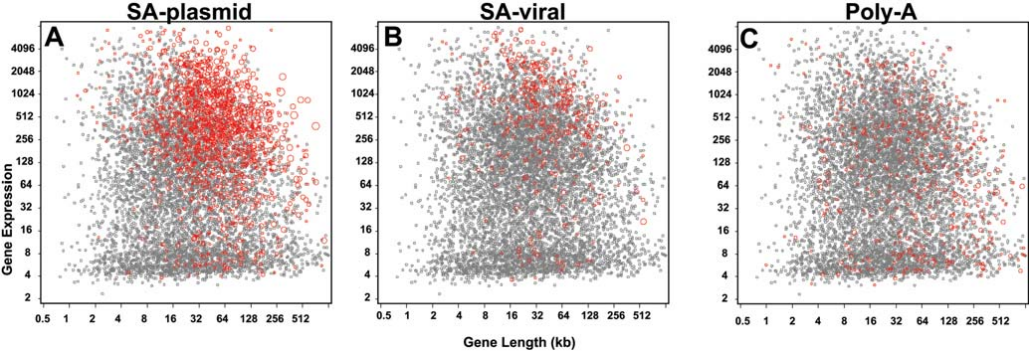
Trapped genes by length and expression. For each vector type, genes were plotted according to their size and level of expression in ESCs. Genes that have been trapped are shown in red. The circle size is proportional to the number of times a gene has been trapped.

**Figure 3:**
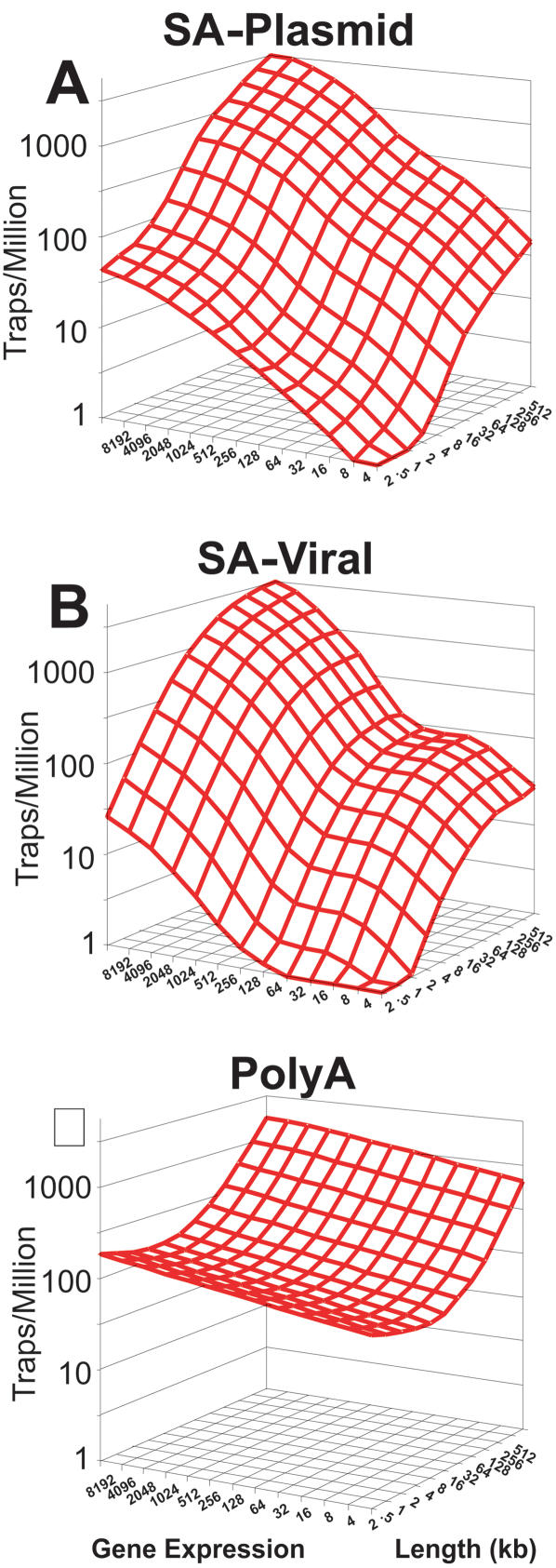
Models of trap likelihood for gene-trap vectors. Models of the likelihood of trapping a gene with particular length (*x*-axis) and expression (*y*-axis) values for each gene-trap event were created through an iterative process, in which outliers (*P*<0.001) were removed before the final model was created. Probability (*z*-axis) is given as events per million traps.

For this study, we focused on three major types of gene-trap vectors (Figure 1), for which enough genes had been trapped to allow robust comparisons. We analyzed 16322 gene-trap cell lines in the public database (www.genetrap.org) (Table 1). We first constructed scatter plots of the trapping frequencies for genes versus known gene length and our expression estimates in E14 ESCs for each vector type (Figure 2). We then used regression modeling to test length and expression simultaneously, so that we could assess the effects of each variable on trapping, independently of the other. For each vector, we fit a regression model to the number of times each gene was trapped as a function of gene length and expression. Spline-based modeling methods were used to accommodate potential nonlinearity in the models of trap likelihood. The expected number of traps for each gene per million trapping events, as predicted by the fitted models, was plotted against a grid of length and expression values (Figure 3).

**Table 1:** Summary of gene trap data sets

Vector	Data set summary			
	Lines	Traps	Genes	% in Genes
SA-plasmid	8410	5857	2683	69.60%
SA-viral	3033	1989	708	65.60%
Poly-A	4879	1748	998	35.80%
All IGTC	49258	29147	5788	59.20%

Lines, number of cell lines in public gene trap database; Traps, number of gene-trap events mapped to a gene; Genes, total number of unique genes trapped; % in Genes, percent of gene-trap events mapped to exon/intron regions (including UTR) of known genes.

The probability that a gene would be trapped with SA-plasmid and SA-viral vectors increased with both gene expression and gene length. SA vectors showed highly statistically significant (P<0.0001) effects due to gene expression (Table 2). In comparison, the poly-A vectors showed much weaker, albeit statistically significant, expression effects (P<0.013). The trapping efficiency of the SA-plasmid and poly-A vectors also increased with gene size (P<0.0001). SA-retroviral vectors exhibited a similar length trend (P<0.0001), but for unknown reasons, these vectors displayed a plateau effect in the largest genes, where trapping likelihood did not increase.

**Table 2:** Hotspot Effects and Model Summary

Vector	Model Summary					Hotspot Effect		
	Modeled Events	Modeled Genes	Expression P Value	Length P Value	Explained Deviance	Hotspot Genes	Traps in Hotspots	% Total Traps
SA-plasmid	3513	1545	<0.0001	<0.0001	34%	26	366	10.42%
SA-retroviral	1187	400	<0.0001	<0.0001	19%	18	358	30.16%
Poly-A	805	442	0.013	<0.0001	6%	9	170	21.12%

Modeled events and genes represent the number of trap events and unique trapped genes considered in the modeling process. *P* values for expression and length represent likelihood ratio significance tests. Explained deviance is analogous to the percent of the variance that is explained in a linear regression model. Hotspots reported as the number of genes that fell outside the hotspot cut-off, the number of trap events in the hotspot gene set, and as the percent of modeled traps in hotspot genes.

The strong effect of expression on trap likelihood is likely due to two factors. First, this effect is an inherent property of antibiotic selection, and differences in the expression trends of endogenous expression-dependent vectors stem from differences between cell-culture and sequencing protocols. Second, the presence of the small expression effect in poly-A vectors, where none is expected, suggests that gene-trap likelihood is, at some level, dependent on transcriptional activity and chromatin structure. Previous studies of retroviral insertion with vectors similar to gene-trap vectors yielded contradictory results concerning the effect of gene-expression level on vector insertion [14]–[16]; however, retroviruses are known to integrate preferentially into transcribed genes, likely owing to the effects of chromatin structure [17].

Although poly-A vectors do not appear to depend substantially on gene expression levels, their use poses additional challenges. Poly-A vectors do not require endogenous regulation of transcription, so there is a potentially greater chance that insertion in a “non-genic” locus could still confer antibiotic resistance. This could account for the diminished proportion of poly-A gene-trap events that can be mapped to a gene (Table 1). In addition, preferential integration at the 3′ end of genes in these vectors is due to nonsense-mediated decay of transcripts of the antibiotic-resistance gene-trap products. This decay typically occurs when the vector inserts upstream of the final intron [18]. As a result, insertion of poly-A-trap vectors at the 3′ end more frequently yields drug-resistant colonies. This bias is worrisome because the likelihood that a gene-trap mutation will cause a null allele decreases as the insertion site moves towards the 3′ end of a gene. Newer poly-A trap vectors may overcome the nonsense-mediated decay issue [18] and could be an attractive alternative to expression-based gene-trapping vectors.

In addition to expression, we found that gene length affected trapping likelihood for all three vectors. This finding was somewhat surprising because certain vector types are thought to insert primarily into the ends of genes and therefore might not be expected to exhibit significant gene-length effects. For example, retroviral vectors preferentially inserted at the 5′ end of genes in one study [9]. Likewise, the poly-A trap vectors included in this study insert preferentially into the 3′ ends of genes [18]. Such preferential insertion could eliminate the effects of overall length. In addition, the first or last introns may be the dominant determinants of this length effect, and that total gene length might not capture this effect. Nevertheless, we found a clear enrichment in the trapping of long genes with all retroviral vectors.

While individual insertion-specific intron length may be of ultimate importance to the length effect observed in this study, measurement of intron size and identification of the intron of insertion are less reliable due to the prominence of alternative splicing and the difficulty of mapping specific gene-trap vector insertion sites. Further characterization of trends affecting the intron of insertion is necessary to better understand the gene-length effect described here.

### Hotspots of vector insertion

Though well known in the field of gene-trapping [9], hotspots are difficult to define rigorously [19]. These loci, in which vector insertion is highly enriched, are of interest not only to the gene-trap community, but also to the gene therapy [19], cancer biology [20], and HIV fields [21]. Prior attempts to define hotspots have likely been confounded by ignoring effects of expression and length that we define here. To identify loci trapped with frequencies elevated beyond those predicted by expression and length, we flagged genes significantly outside the model prediction space and defined them as hotspots. We used an iterative fitting process to identify these genes separately for each vector (hotspots listed in Table S2).

Each vector type had a unique set of hotspots, with marked differences based upon the method of vector delivery (plasmid or retroviral), as shown in Table 2. Hotspot insertions were more frequent with SA-retroviral retroviral vectors (30% of total traps) than with SA-plasmid vectors (10% of total traps). Poly-A vectors showed a smaller hotspot effect (21% of total traps) than other retroviral vectors. These proportions could underestimate the actual number of genomic hotspots, as we only considered well-defined genes.

This increased presence of hotspots in retroviral-based gene trapping could reflect the tendency of retroviral insertional machinery to interact with specific sites in the genome [19], [22]–[24]. The mechanism driving hotspots with plasmid vectors is less well understood but might involve genomic regions with high recombination frequencies and high rates of double-stranded break repair [25]–[27]. The quantitative method of identifying hotspots used here may help future investigations to identify and characterize cellular and genomic factors that underlie insertional hotspots.

### Summary of gene-trap likelihood models

The ability of these expression and length-dependent models to explain trapping probabilities was quantified by the percent reduction in deviance compared to a null model with no covariates in the datasets after hot-spot removal, analogous to the use of R-squared in linear regression models. The percent reduction in deviance was greater for SA-plasmid and SA-retroviral models (34% and 19%, respectively) than for the poly-A model (6%). For the expression-dependent SA vectors, these models explain a substantial proportion of the deviance. The low explanatory ability of the poly-A vector model reflects the relatively lower effect of expression on gene-trap likelihood, and to some extent the smaller effect of length.

Regardless of whether a gene has been inactivated by gene trapping in our experimental data, our models can predict the likelihood that a gene will be trapped in a single trapping event. These predictions serve as “trapping scores” for each gene. The raw trapping scores were corrected for the effects of both hotspots and gene-trap events that could not be mapped to a gene. The corrected scores, reported for the 7435 well-defined genes included in our dataset in Table S2, allow the overall trapping efficiencies of different vectors to be compared.

To validate our model, we compared the expected number of traps from the SA-retroviral model with the observed number of traps in gene-trap cell lines produced by Lexicon Genetics [28]. This validation set contained 48,809 cell lines from Lexicon Genetics that could be annotated to our gene set (data in Table S2). The Spearman's correlation coefficient for the comparison was 0.429 (P<0.005). This level of concordance gives us further confidence that gene-trap likelihood was successfully modeled.

Although these models describe a significant proportion of the variance in trap likelihood between mouse genes, other factors undoubtedly contribute to trap likelihood, and therefore, trapping scores should be interpreted on a limited scope for individual genes. For instance, the expression-based vectors used in this study fail to trap secreted and membrane-bound proteins (data not shown). Other genomic factors that control vector insertion, transcription, splicing, translation and protein localization all likely play some role in determining trap likelihood. Further examination of genes where the number of observed traps departs from model predictions may help identify other important mechanisms affecting insertional mutagenesis.

Our models have other limitations relating to experimental and modeling constraints. The restricted number of total genes for which high-confidence annotation and ESC expression data were available reduced our dataset size. We also were limited to using expression measurements from a single ESC line, and different global expression states potentially exist between the different ESC lines used in gene trapping. These constraints may affect the accuracy of our models, contributing to the overall noise, and these effects may be stronger at the end of the length and expression scales, where there are fewer data points.

### Conclusion

Our findings offer a more complete understanding of factors governing the accessibility of genes to trapping. We report the first formal testing of the effects of gene expression and gene length on trapping likelihood. While the effects of expression in SA-trap vectors is confirmatory, the detection of an expression effect in poly-A vectors is an important finding and matches previous reports of a role of transcriptional state in vector insertion likelihood. The length effect reported for all vectors described in this study is a novel finding that requires further characterization to understand the relative importance of the underlying biology. In addition the identification of expression and length-independent insertional hotspots is an important result and could benefit fields other than gene-trapping. Ideally, the empirically quantified relationships we provide here can be generalized to all genes in the mouse genome. Mutagenesis in mouse and human ESCs will continue to evolve with new and more powerful techniques, and the results from this initial analysis will hopefully aid future mammalian gene mutagenesis efforts.

## Materials and Methods

### Gene-trap data

Data for gene-trap cell lines were generated with the International Gene Trap Consortium (IGTC) identification and annotation pipeline [29]. Annotations were obtained by genome and transcript-based homology searching. Publicly available gene-trap cell lines included in the IGTC database were used for the gene-trap data sets, and all gene-trap cell-line sequences used in our analysis can be found in the NCBI Genome Survey Sequence database [30].

Because numerous gene-trap vectors were used to create the cell lines represented by the IGTC, we chose three representative vector groups for analysis: the plasmid pGTlxf series (SA-plasmid), the retroviral FlEx vectors (SA-retroviral), and a combination of poly-A trap vectors (poly-A). The SA-plasmid vectors were produced by BayGenomics (http://baygenomics.ucsf.edu). The SA-viral vectors are a conditional system used by the German Gene Trap Consortium (http://www.tikus.gsf.de) [31]. Poly-A cell lines are from the Centre for Modeling Human Disease gene-trap project (http://www.cmhd.ca/genetrap) [13]. Poly-A vectors designed to take advantage of nonsense-mediated decay [18] were not included in this analysis. Exon trap vectors are similar to SA-trap vectors but depend on direct, in-frame integration into the open-reading frame of a gene [32]; however, these vectors behave similarly to SA vectors due to cryptic splicing [33] and were therefore omitted from this study. Secretory vectors containing transmembrane signal sequences [34], [35] were also excluded. Vector maps are available on each gene-trap resource website. More information on gene-trap data is available on the IGTC website (http://www.genetrap.org).

### Gene Data

Gene length and annotation data were from mouse build 36 of the Ensembl database [36]. Length was computed as the full transcribed genomic region, including the UTR when present. This analysis used a set of 7435 well-characterized genes (Table S2) for which complete sequence data were available. Similar sets of “sentinel genes” have been used in analyses of gene-trap data [28]. For this study, this set includes known genes annotated to a Mouse Genome Informatics (MGI) symbol and an Entrez Gene ID and not primarily identified by Riken clones. Single-exon genes were omitted from the model, because they lack of a splice donor site necessary for proper function of SA and poly-A trap vectors.

### Expression Data

For all gene-expression data, mouse E14 ESCs were prepared as described [37] (http://www.baygenomics.ucsf.edu). For GeneChip studies, we performed four biological replicates using Affymetrix 430 2.0 arrays, and RNA samples were prepared as described by the manufacturer (Affymetrix, Sunnyvale, CA). The 430 2.0 GeneChip contains 45,101 probe sets, including 9242 probe sets that were unambiguously mapped to a single Ensembl identification. Only probes marked as type “_at” were used for the final analysis, because we had the highest confidence of proper hybridization in these probe sets. All other probe sets were discarded, because the probes may cross-hybridize to mRNA products of other genes (Affymetrix). We selected the probe set with maximal expression when there was more than one representing a single gene.

Expression values were calculated with GCRMA (v. 1.1.5; http://www.bioconductor.org), a method that purports to give good estimates of expression in the entire expression spectrum [38]. While on/off calls and removal of genes based on a low signal-to-noise ratio may allow elimination of some spurious expression results, use of GCRMA and full data inclusion were necessary to model the likelihood of trapping, especially at the lower boundaries of expression. Expression values for a subsample of genes from the same cell type in the same tissue-culture conditions were confirmed in TaqMan quantitative RT-PCR experiments [37]. Raw and normalized data for these experiments can be accessed using GEO series accession GSE8128.

### Analysis

Spline-based methods were used in multinomial regression models, with gene length and expression as model predictors and the number of trap events in each gene as the outcome. Knot placement for splines was based on gene-distribution percentiles. Models were iteratively fitted to genes remaining at each round after cumulative removal of hotspot genes, defined as genes with an observed trapping frequency far above the expected frequency. We identified such genes, using a cut-off of P<0.001 and re-ran the analyses without them. These probabilities were calculated by using the Poisson approximation to the binomial distribution with a large number of trials and a low probability of success and were corrected for the estimated model overdispersion. We then re-fit the model with the hotspots deleted, repeating the process until no additional hotspots were identified. The level of significance 0.001 *P* value was selected to be conservative in the culling of genes that did not fit the model, as we wanted to limit the number of genes removed to only those that far exceeded predicted trap likelihood. *P* values for the length and expression effects in the final models are reported, and deviance that can be explained for each model was computed.

Trapping scores were computed directly from the fitted model as the predicted probability of trapping, and corrected by multiplying the proportion of events that trapped a modeled gene rather than a hotspot or gene-trap event that could not be mapped to a gene. Statistical analysis was performed with SAS (SAS Institute, Cary, NC) and the R statistical environment (http://www.r-project.org).

### Model Specification

Because each experiment (trap event) selects one of a known set of genes that could be trapped, the data fit the statistical framework of multinomial regression. Let *n* = 1 to *N* index experiments that trapped a gene. For each experiment, we assumed that the probability that gene *j* is the one that is trapped is a function of covariates. Let **x** be the matrix with a row for each gene and a column for each covariate. A multinomial model for which gene is trapped in each experiment is then defined by: 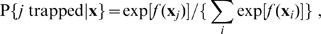
(1)where the sum in the denominator is over all genes that might be trapped, *f* is a function of the covariates, and **x** *_i_* is the vector of covariates for gene *i*. For example, a simple linear model with two covariates would be f(**x** *_i_*) = β_1_ *x_i1_*+β_2_ *x_i2_*. Here we restrict attention to genes whose length and expression are known and to the experiments where one of these genes was trapped. Letting *j* denote the gene trapped in the *n*th experiment, we can write the log-likelihood (up to a constant that does not depend on the covariates) for experiment *n* as: 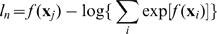
(2)Letting *h_i_* denote the number of times gene *i* was trapped, we can then write the log-likelihood for the entire set of experiments as: 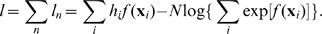
(3)


For any given parametric form for the function *f*, we can estimate the parameters by finding those that maximize this log-likelihood, with the general optimization features of the NLMIXED procedure in SAS. For both covariates (gene length and expression), we applied logarithmic transformations and then used cubic parametric splines [39], choosing among models with different degrees of freedom with the Akaike information criterion [40] adjusted for overdispersion [41]. We assumed that the effects of these two covariates were additive, *f*(**x** *_i_*) = *f_1_*(*x_i1_*)+*f_2_*(*x_i2_*). Adding interaction terms did not substantially improve fits to the data.

To calculate a fitted probability of trapping for each gene, we used equation (1) with the best-fitting parameters for *f*_1_ and *f*_2_

## Supporting Information

Table S1 Compares the expression as measured using RT-PCR with expression estimates derived using GCRMA methods.
(0.03 MB XLS)

Table S2 Summarizes the dataset used for this analysis. Included are the length and expression values for genes included in the analysis, number of traps in each gene by gene-trap vector, and the derived trap score for each gene. Hotspots genes are tagged in the trap score column for each vector. Omniback gene-trap events, used here as a validation set, are listed in the final column.
(1.60 MB XLS)
